# Evaluation of the prognostic relevance of the recommended minimum number of lymph nodes in colorectal cancer—a propensity score analysis

**DOI:** 10.1007/s00384-021-03835-8

**Published:** 2021-01-16

**Authors:** Michaela Ramser, Leonard A. Lobbes, Rene Warschkow, Carsten T. Viehl, Johannes C. Lauscher, Raoul A. Droeser, Christoph Kettelhack, Markus Zuber, Benjamin Weixler

**Affiliations:** 1 Clarunis Visceral Surgery Center, St. Clara Hospital & University Hospital Basel, Basel, Switzerland; 2 Department of Surgery, Kantonsspital Olten, Olten, Switzerland; 3 Department of General, Visceral, and Vascular Surgery, Charité University Hospital, Campus Benjamin Franklin, Berlin, Germany; 4 Department of Surgery, Kantonsspital St. Gallen, St. Gallen, Switzerland; 5 Institute of Medical Biometry and Informatics, University of Heidelberg, Heidelberg, Germany; 6 Department of Surgery, Spitalzentrum Biel/Bienne, Biel/Bienne, Switzerland

**Keywords:** Colorectal cancer, Lymph nodes, Overall survival, Disease-free survival, Propensity score analysis

## Abstract

**Purpose:**

Nodal status in colorectal cancer (CRC) is an important prognostic factor, and adequate lymph node (LN) staging is crucial. Whether the number of resected and analysed LN has a direct impact on overall survival (OS), cancer-specific survival (CSS) and disease-free survival (DFS) is much discussed. Guidelines request a minimum number of 12 LN to be analysed. Whether that threshold marks a prognostic relevant cut-off remains unknown.

**Methods:**

Patients operated for stage I–III CRC were identified from a prospectively maintained database. The impact of the number of analysed LN on OS, CSS and DFS was assessed using Cox regression and propensity score analysis.

**Results:**

Of the 687 patients, 81.8% had ≥ 12 LN resected and analysed. Median LN yield was 17.0 (IQR 13.0–23.0). Resection and analysis of ≥ 12 LN was associated with improved OS (HR = 0.73, 95% CI: 0.56–0.95, *p* = 0.033), CSS (HR 0.52, 95% CI: 0.31–0.85, *p* = 0.030) and DFS (HR = 0.73, 95% CI: 0.57–0.95, *p* = 0.030) in multivariate Cox analysis. After adjusting for biasing factors with propensity score matching, resection of ≥ 12 LN was significantly associated with improved OS (HR = 0.59; 95% CI: 0.43–0.81; *p* = 0.002), CSS (HR = 0.34; 95% CI: 0.20–0.60; *p* < 0.001) and DFS (HR = 0.55; 95% CI: 0.41–0.74; *p* < 0.001) compared to patients with < 12 LN.

**Conclusion:**

Eliminating biasing factors by a propensity score matching analysis underlines the prognostic importance of the number of analysed LN. The set threshold marks the minimum number of required LN but nevertheless represents a cut-off regarding outcome in stage I–III CRC. This analysis therefore highlights the significance and importance of adherence to surgical oncological standards.

## Introduction

Colorectal cancer is still one of the most common malignancies and a leading cause of cancer-related death [1, 2]. Surgical resection following oncologic principles including systematic lymphadenectomy is the treatment of choice. The resection and analysis of at least 12 lymph nodes (LN) is recommended by most national and international guidelines [3–7]. The lower limit of 12 LN was set in 1990 by the World Congress of Gastroenterology [8]. However, this was rather a randomly selected numerical value, with no higher-level evidence available at the time. Whether that threshold marks a prognostic relevant cut-off remains unknown.

Over the years, the level of evidence improved, that in fact the number of LN was a relevant prognostic factor, preventing understaging by missed positive LN [9–12]. Since the presence of nodal metastases is still one of the most important prognostic factors in colorectal cancer, an adequate number of analysed LN is crucial [9, 13].

Given the nature of the problem, to our knowledge, no standard randomised trial addressing the validity of the set threshold of 12 LN is available. Additionally, based on the existing evidence regarding the number of LN to be resected, conduction of a randomised study is no longer ethically justifiable. A propensity score matching analysis, which accounts for possible bias in non-randomised studies by eliminating the different distribution of observed variables between two groups, is one of the best ways to answer this question instead.

The aim of this study was to test whether the threshold of 12 resected and analysed LN actually marks a prognostic relevant cut-off level regarding cancer-specific outcome in patients operated for colorectal cancer, using propensity score matching.

## Methods

**Fig. 1:**
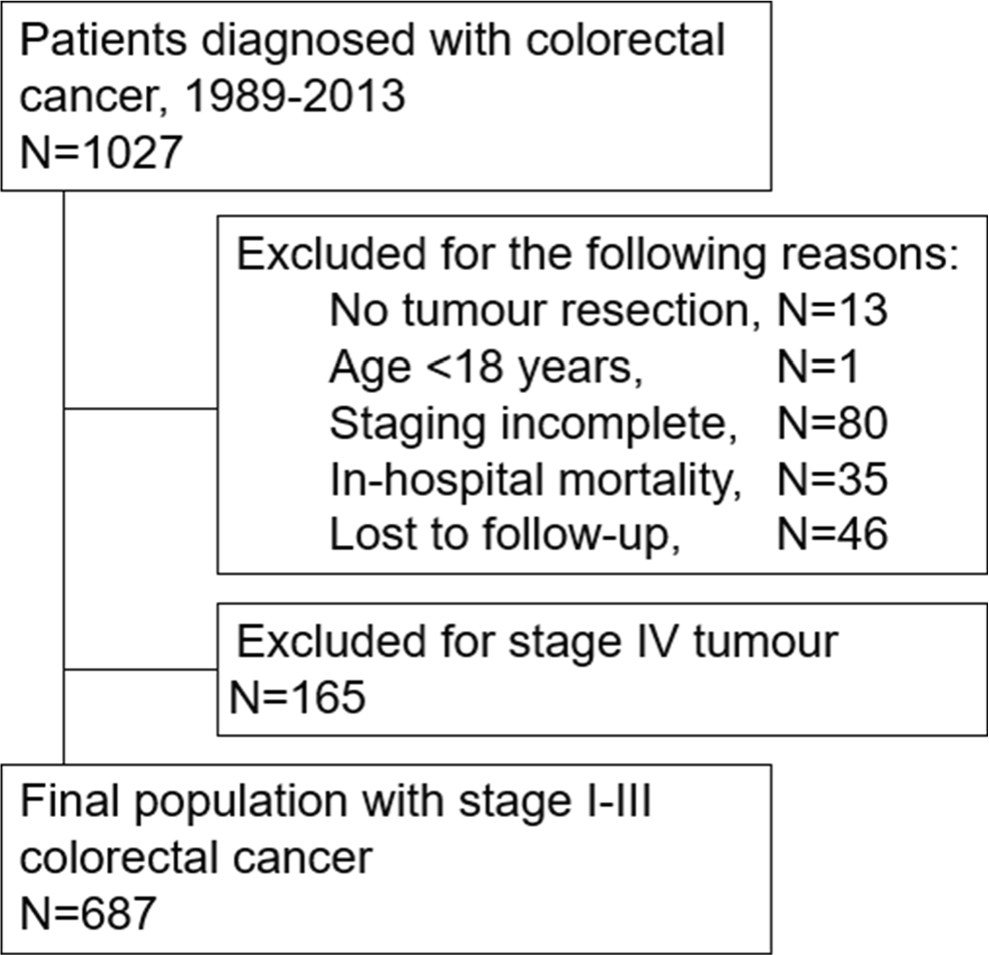
Flow chart of the selection process

Data for this retrospective study were extracted from the prospectively maintained cancer registry from three university-affiliated institutions in Switzerland: the Department of Surgery at the University Hospital Basel, the Cantonal Hospital Olten and the Hospital Center Biel/Bienne. Between 1989 and 2013, a total of 1027 patients were treated for colorectal cancer at the three institutions. Patients with stage IV disease (165/852, 19.4%) were excluded from further analysis (Fig. 1). A total of 687 patients with stage I–III colorectal cancer were finally eligible for statistical analysis. For uniform histopathological tumour staging, all cases were retrospectively reassessed and staged according to the 7th TNM classification system at the time of follow-up data collection [14].

Data collection and analysis of the cancer registry was approved by the local ethical committee (EK 120/13 and clinicaltrials.gov identifier: NCT00826579). Approval of data collection was obtained prior to surgery (1989–2005), and a consent was obtained by letters of enquiry (2006–2013). Follow-up data and information on mortality were collected from the respective treating general practitioner, hospital records and the bureau of vital statistics.

(Neo)adjuvant radiation and/or chemotherapy was routinely offered to patients with a qualifying tumour stage or risk factors and in line with the respective recommendations and guidelines at the time.

### Statistical analyses

Statistical analyses were performed using the R statistical software (www.r-project.org). A two-sided *p* value < 0.05 was considered statistically significant. Continuous data are expressed as means ± standard deviation. For comparing proportions, chi-square statistics and, for comparing continuous variables, *t* tests were used. For regression analysis, likelihood ratio tests were performed. After descriptive analysis, the bias concerning the number of resected and analysed LN (< 12 vs ≥ 12) was assessed by logistic regression. Unadjusted and risk-adjusted Cox regression with robust sandwich variance estimators and stratification for the study centre were performed to assess the putative prognostic impact of the number of resected and analysed LN on overall (OS), cancer-specific (CSS) and disease-free survival (DFS). Multivariable Cox regression models were complemented by a backward variable selection based on the Akaike’s information criterion. To optimally control for possible confounding differences in baseline characteristics, a bipartite weighting propensity score analysis was performed using the “matching” R package [15–19]. The baseline risk profiles of the matched patient characteristics were compared by conditional logistic regressions to assure that no major differences persisted. The prognostic value of < 12 vs ≥ 12 resected and analysed LN for OS, CSS and DFS was finally assessed in a stratified Cox regression analysis applying the subclasses and the weights obtained by the propensity score analysis.

The diagnostic accuracy of different cut-offs established by the numbers of resected and analysed LN was assessed by multimodel inference [20, 21] using resampling methods. The diagnostic accuracy was assessed in terms of the Akaike’s information criterion (AIC) and the integrated area under the curve (iAUC) as described by Chambless and Diao [22]. For the AIC, multivariable Cox regression models with cut-offs between 5 and 20 for the number of resected and analysed LN with adjustment for colon/rectum, tumour stage, age and year of operation were fitted in resampled study data for OS, CSS and DFS survival. For each cut-off, 10,000 bootstrap samples were drawn, and Cox regression models were fitted for each survival measure in each of these samples. For each cut-off, the median of the AIC was estimated. For the iAUC, the study data were 10,000 times randomly divided into a training and a test data set. Multivariable Cox regression models with cut-offs between 5 and 20 for the number of resected and analysed LN with adjustment for colon/rectum, tumour stage, age and year of operation were fitted in the training data sets for OS, CSS and DFS. The fits obtained in the training data set were applied in the similar Cox models in the test data set. Based on the latter models, the iAUC was estimated using the R library “AUCsurv”. Finally, the median of the iAUC was estimated for each cut-off.

## Results

### Patient characteristics

Demographic patient information and histopathological tumour details are shown in Table 1. Overall, 22.9% of tumours were UICC stage I, 38.9% stage II and 38.3% stage III.

**Table 1:** Demographic information and histopathological tumour details for all patients

Baseline						Multivariable logistic regression		Baseline after propensity score matching		
		Total	< 12 LN	≥ 12 LN	*p* value	Odds ratio (95% CI)	*p* value^C)^	< 12 LN	≥ 12 LN	*p* value^D)^
		*n* = 687	*n* = 125	*n* = 562				*n* = 122	*n* = 442	
Tumour localisation	Colon	475 (69.1%)	70 (56.0%)	405 (72.1%)	< 0.001 A)	Reference	0.317	79.8 (65.4%)	305 (69.0%)	0.986
	Rectum	212 (30.9%)	55 (44.0%)	157 (27.9%)		0.76 (0.46–1.30)		42.2 (34.6%)	137 (31.0%)	
UICC stage	I	157 (22.9%)	48 (38.4%)	109 (19.4%)	< 0.001 B)	Reference	0.002	27.4 (22.5%)	103 (23.3%)	1.000
	IIA	221 (32.2%)	30 (24.0%)	191 (34.0%)		2.58 (1.48–4.54)		27.3 (22.4%)	149 (33.7%)	
	IIB	37 (5.4%)	3 (2.4%)	34 (6.0%)		3.80 (1.19–17.09)		3.9 (3.2%)	21 (4.8%)	
	IIC	9 (1.3%)	2 (1.6%)	7 (1.2%)		1.66 (0.34–12.16)		1.4 (1.1%)	7 (1.6%)	
	IIIA	42 (6.1%)	11 (8.8%)	31 (5.5%)		1.02 (0.44–2.47)		9.7 (8.0%)	31 (7.0%)	
	IIIB	165 (24.0%)	29 (23.2%)	136 (24.2%)		1.88 (1.01–3.53)		47.3 (38.8%)	118 (26.7%)	
	IIIC	56 (8.2%)	2 (1.6%)	54 (9.6%)		7.53 (2.02–49.15)		5 (4.1%)	13 (2.9%)	
Grading	G1	22 (3.2%)	7 (5.6%)	15 (2.7%)	0.194 B)	Reference	0.402	4.7 (3.8%)	13 (2.9%)	1.000
	G2	532 (77.4%)	97 (77.6%)	435 (77.4%)		2.06 (0.70–5.63)		105.8 (86.7%)	352 (79.6%)	
	G3	133 (19.4%)	21 (16.8%)	112 (19.9%)		1.86 (0.58–5.68)		11.5 (9.4%)	77 (17.4%)	
Year of operation	1989–1999	161 (23.4%)	48 (38.4%)	113 (20.1%)	< 0.001 B)	Reference	< 0.001	22.6 (18.6%)	113 (25.6%)	0.998
	2000–2003	207 (30.1%)	40 (32.0%)	167 (29.7%)		1.19 (0.69–2.06)		34.5 (28.3%)	121 (27.4%)	
	2004–2007	206 (30.0%)	21 (16.8%)	185 (32.9%)		3.07 (1.68–5.76)		32 (26.2%)	130 (29.4%)	
	2008–2013	113 (16.4%)	16 (12.8%)	97 (17.3%)		2.78 (1.42–5.66)		32.8 (26.9%)	78 (17.6%)	
Sex	Female	297 (43.2%)	42 (33.6%)	255 (45.4%)	0.016 A)	Reference	0.026	39.5 (32.4%)	191 (43.2%)	0.797
	Male	390 (56.8%)	83 (66.4%)	307 (54.6%)		0.61 (0.39–0.94)		82.5 (67.6%)	251 (56.8%)	
Age	< 60	121 (17.6%)	21 (16.8%)	100 (17.8%)	0.139 B)	Reference	0.531	27.1 (22.2%)	80 (18.1%)	0.997
	60–69	176 (25.6%)	42 (33.6%)	134 (23.8%)		0.70 (0.36–1.32)		28.6 (23.4%)	114 (25.8%)	
	70–79	208 (30.3%)	35 (28.0%)	173 (30.8%)		0.79 (0.39–1.54)		47.8 (39.2%)	123 (27.8%)	
	≥ 80	182 (26.5%)	27 (21.6%)	155 (27.6%)		1.03 (0.49–2.12)		18.5 (15.2%)	125 (28.3%)	
Centre	KSO	84 (12.2%)	2 (1.6%)	82 (14.6%)	< 0.001 A)	Reference	< 0.001	6.3 (5.2%)	9 (2.0%)	0.961
	SZB	36 (5.2%)	3 (2.4%)	33 (5.9%)		0.26 (0.03–1.68)		10.5 (8.6%)	22 (5.0%)	
	USB	567 (82.5%)	120 (96.0%)	447 (79.5%)		0.11 (0.02–0.38)		105.2 (86.2%)	411 (93.0%)	
Cancer resection	Elective	629 (91.6%)	117 (93.6%)	512 (91.1%)	0.364 A)	Reference	0.954	117.9 (96.6%)	404 (91.4%)	0.980
	Emergency	58 (8.4%)	8 (6.4%)	50 (8.9%)		0.97 (0.41–2.54)		4.1 (3.4%)	38 (8.6%)	
Tumour perforation	No	634 (92.3%)	116 (92.8%)	518 (92.2%)	0.812 A)	Reference	0.806	111.5 (91.4%)	404 (91.4%)	0.973
	Yes	53 (7.7%)	9 (7.2%)	44 (7.8%)		0.90 (0.39–2.22)		10.5 (8.6%)	38 (8.6%)	
Chemotherapy	No	460 (67.0%)	86 (68.8%)	374 (66.5%)	0.628 A)	Reference	0.145	74.4 (61.0%)	303 (68.6%)	0.940
	Yes	227 (33.0%)	39 (31.2%)	188 (33.5%)		1.60 (0.85–3.09)		47.6 (39.0%)	139 (31.4%)	
Radiation therapy	No	604 (87.9%)	100 (80.0%)	504 (89.7%)	0.003 A)	Reference	0.056	101.7 (83.4%)	388 (87.8%)	0.932
	Yes	83 (12.1%)	25 (20.0%)	58 (10.3%)		0.46 (0.21–1.02)		20.3 (16.6%)	54 (12.2%)	

Patients are grouped according to the number of retrieved lymph nodes. Multivariable logistic regression model and patient information of the remaining population after the matching procedure for the propensity score analysis. The patients are grouped according to the number of retrieved lymph nodes

Results presented in *n* (percent %) and odds ratio (95% CI). *UICC* Union for International Cancer Control, *LN* lymph node, *T* tumour stage, *N* nodal stage; 95% CI, 95% confidence interval

A) Chi-squared test

B) Mann-Whitney *U* test

C) Likelihood ratio tests in multivariable logistic regression

D) Likelihood ratio tests in univariable conditional logistic regression analyses

A majority of the patients were male (56.8%). The larger proportion of tumours were localised in the colon, while 30.9% were rectal cancers. Overall, 91.6% of tumours were resected in an elective operation.

In 81.8% of all cases, ≥ 12 LN were resected and analysed, while the median number was 17.0 (interquartile range (IQR) 13.0–23.0). The number of LN per patient differed significantly between patients with stage I, II and III colorectal cancer when analysed in a multivariable logistic regression model (*p* < 0.001) (Table 1).

The data on neoadjuvant and adjuvant therapy shows that overall, 12.1% of patients received radiation therapy and 33.0% chemotherapy (Table 1).

### Number of resected and analysed lymph nodes

For further analysis, the population was divided into two groups with patients with less than 12 analysed LN (< 12) and 12 or more LN (≥ 12). Demographic information and tumour details of patients in the two groups are shown in Table 1. Over the years, the proportion of patients with more analysed LN increased. A difference in distribution between the two groups was also observed for tumour stage, tumour localisation, sex, the treating centre and indication for radiation therapy. On the other hand, age, grading and elective vs emergency operations were equally distributed between patients with fewer or more analysed LN. All of the above variables, expect for the tumour localisation and radiation therapy, remained significantly unequally distributed between the two groups in a multivariable logistic regression model (Table 1).

The 5-year survival rates for OS and DFS increased when more LN were analysed. Five-year OS rates for patients with < 12 LN was 65.8% (95% confidence interval (CI): 57.7–75.1) and 66.3% (95% CI: 62.1–70.8) for patients with ≥ 12 LN. Five-year DFS for patients with < 12 LN was 53.2% (95% CI: 44.9–63.2) and 58.8% (95% CI: 54.5–63.5) for patients with ≥ 12 LN.

### Overall survival—uni- and multivariate analyses

In univariate analysis, higher age at the time of operation, higher tumour stage and higher tumour grading were risk factors for decreased overall survival (OS), while localisation in the rectum and administration of chemo- and radiation therapy were factors for an improved OS. In multivariate analysis, higher age, higher tumour stage as well as needing radiation therapy were independent risk factors for a decreased OS, while localisation in the rectum and a higher number of resected and analysed LN (≥ 12 LN) were related to an improved OS (Table 2). The association between retrieval of ≥ 12 LN and improved OS was confirmed in stepwise variable selection (HR = 0.73, 95% CI: 0.56–0.96, *p* = 0.033).

**Table 2:** Uni- and multivariate analyses on overall survival, cancer-specific survival and disease-free survival

	Overall survival				Cancer-specific survival				Disease-free survival			
	Univariate analysis^A)^		Multivariate analysis^B)^		Univariate analysis^A)^		Multivariate analysis^B)^		Univariate analysis^A)^		Multivariate analysis^B)^	
	HR (95% CI)	P^C)^	HR (95% CI)	P^C)^	HR (95% CI)	P^C)^	HR (95% CI)	P^C)^	HR (95% CI)	P^C)^	HR (95% CI)	P^C)^
Retrieved LN												
< 12	Reference	0.779	Reference	0.033	Reference	0.720	Reference	0.030	Reference	0.777	Reference	0.030
12	0.96 (0.74–1.26)		0.73 (0.56–0.96)		0.90 (0.53–1.54)		0.52 (0.31–0.85)		0.96 (0.74–1.25)		0.73 (0.57–0.95)	
Tumour localisation												
Colon	Reference	< 0.001	Reference	0.014	Reference	0.030	Reference	0.064	Reference	0.010	Reference	0.148
Rectum	0.66 (0.51–0.84)		0.70 (0.51–0.95)		0.59 (0.36–0.96)		0.54 (0.24–1.19)		0.74 (0.59–0.94)		0.82 (0.61–1.09)	
UICC stage												
I	Reference	< 0.001	Reference	< 0.001	Reference	< 0.001	Reference	< 0.001	Reference	< 0.001	Reference	< 0.001
IIa	1.35 (0.99–1.85)		1.27 (0.94–1.72)		2.66 (1.08–6.55)		2.31 (0.92–5.77)		1.42 (1.06–1.91)		1.38 (1.03–1.86)	
IIb/IIc	1.43 (0.84–2.44)		1.37 (0.88–2.15)		3.43 (1.04–11.32)		3.17 (0.99–10.09)		1.47 (0.91–2.38)		1.49 (0.97–2.29)	
IIIa	1.03 (0.60–1.76)		1.09 (0.64–1.84)		3.26 (1.10–9.65)		2.84 (1.02–7.92)		1.11 (0.68–1.83)		1.13 (0.69–1.86)	
IIIb/IIIc	2.37 (1.75–3.21)		2.68 (1.98–3.63)		7.38 (3.19–17.07)		7.94 (3.41–18.46)		2.28 (1.71–3.03)		2.60 (1.95–3.46)	
Grading												
G1	Reference	0.045	–	–	Reference	0.075	–	–	Reference	0.007	–	–
G2	0.90 (0.50–1.62)		–		2.18 (0.31–15.39)		–		0.93 (0.53–1.63)		–	
G3	1.29 (0.68–2.42)		–		3.79 (0.52–27.78)		–		1.42 (0.77–2.60)		–	
Year of operation												
1989–1999	Reference	0.200	–	–	Reference	0.856	–	–	Reference	0.058	Reference	0.109
2000–2003	1.07 (0.80–1.44)		–		1.25 (0.67–2.36)		–		1.14 (0.85–1.52)		1.16 (0.86–1.53)	
2004–2007	1.20 (0.88–1.64)		–		1.13 (0.60–2.13)		–		1.39 (1.04–1.86)		1.30 (0.96–1.75)	
2008–2013	1.53 (1.04–2.26)		–		1.36 (0.63–2.91)		–		1.53 (1.08–2.19)		1.58 (1.12–2.22)	
Sex												
Female	Reference	0.699	–	–	Reference	0.771	–	–	Reference	0.743	–	–
Male	0.96 (0.76–1.20)		–		1.07 (0.68–1.68)		–		1.04 (0.84–1.28)		–	
Age												
< 60 years	Reference	< 0.001	Reference	< 0.001	Reference	< 0.001	Reference	< 0.001	Reference	< 0.001	Reference	< 0.001
60–69 years	1.42 (0.91–2.20)		1.50 (0.95–2.35)		0.57 (0.29–1.16)		0.61 (0.29–1.28)		1.24 (0.82–1.88)		1.28 (0.83–1.96)	
70–79 years	2.63 (1.73–4.01)		2.99 (1.93–4.66)		1.33 (0.72–2.44)		1.47 (0.74–2.89)		2.12 (1.44–3.13)		2.25 (1.50–3.38)	
80 years	6.04 (4.06–8.99)		7.35 (4.70–11.49)		3.39 (1.82–6.32)		4.08 (1.93–8.63)		3.86 (2.66–5.61)		4.30 (2.86–6.46)	
Cancer resection												
Elective	Reference	0.061	–	–	Reference	0.040	–	–	Reference	0.233	–	–
Emergency	1.43 (1.01–2.02)		–		2.08 (1.08–4.04)		–		1.25 (0.88–1.75)		–	
Tumour perforation												
No	Reference	0.492	–	–	Reference	0.042	Reference	0.131	Reference	0.405	–	–
Yes	1.15 (0.79–1.66)		–		2.01 (1.09–3.72)		1.69 (0.89–3.19)		1.17 (0.81–1.70)		–	
Chemotherapy												
No	Reference	0.004	–	–	Reference	0.839	–	–	Reference	0.047	–	–
Yes	0.70 (0.54–0.90)		–		1.05 (0.67–1.64)		–		0.80 (0.63–1.01)		–	
Radiation therapy												
No	Reference	0.017	Reference	0.041	Reference	0.867	Reference	0.097	Reference	0.116	Reference	0.116
Yes	0.66 (0.47–0.94)		1.62 (1.02–2.57)		0.95 (0.53–1.71)		2.08 (0.78–5.58)		0.77 (0.55–1.09)		1.40 (0.91–2.16)	

*UICC* Union for International Cancer Control, *LN* lymph node, *HR* hazard ratio, *95% CI* 95% confidence interval

A) Univariable cox regression analysis for each of the prognosticators

B) Multivariable cox regression analysis after backward variable selection from full models based on the Akaike’s information criterion (AIC)

C) Likelihood ratio tests

### Cancer-specific survival—uni- and multivariate analyses

In univariate analysis, higher age, higher tumour stage and emergency surgery were risk factors for decreased cancer-specific survival (CSS), while tumour localisation in the rectum was associated with an improved CSS. In multivariate analysis, higher age and higher tumour stage were independent risk factors for a decreased CSS, while a higher number of resected and analysed LN (≥ 12 LN) were related to an improved CSS (Table 2). The association between retrieval of ≥ 12 LN and improved CSS was confirmed in stepwise variable selection (HR = 0.52, 95% CI: 0.31–0.85, *p* = 0.030).

### Disease-free survival—uni- and multivariate analyses

In the univariate analysis, higher age, higher tumour stage and tumour grading were risk factors for a shorter disease-free survival (DFS), while tumour localisation in the rectum and chemotherapy improved DFS. In the multivariate analysis, higher age and higher tumour stage were independent risk factors for a decreased DFS, while ≥ 12 resected and analysed LN was an independent factor improving DFS (Table 2). In the stepwise variable selection procedure, the retrieval of  ≥ 12 LN was selected as a significant predictor for improved DFS (HR = 0.73, 95% CI: 0.57-0.95, p=0.030).

### Propensity score analysis

Before the matching, the propensity score for patients with < 12 LN was 0.709 ± 0.152 compared to 0.808 ± 0.124 in patients with ≥ 12 LN (*p* < 0.001), thus indicating a strong and clinically relevant bias regarding the observed patient characteristics in the two groups (Table 1). After the matching procedure, the propensity score was the same in the two patient groups, 0.808 ± 0.124 for patients with < 12 LN compared to 0.808 ± 0.124 for patients with ≥ 12 LN (*p* = 0.992), thus indicating no persisting bias regarding the observed patient characteristic in the two groups. Three patients of the < 12 LN group and 120 patients of the ≥ 12 LN group could not be matched, resulting in a population of 564 patients (Table 1).

### Survival analyses after the propensity score matching

**Fig. 2:**
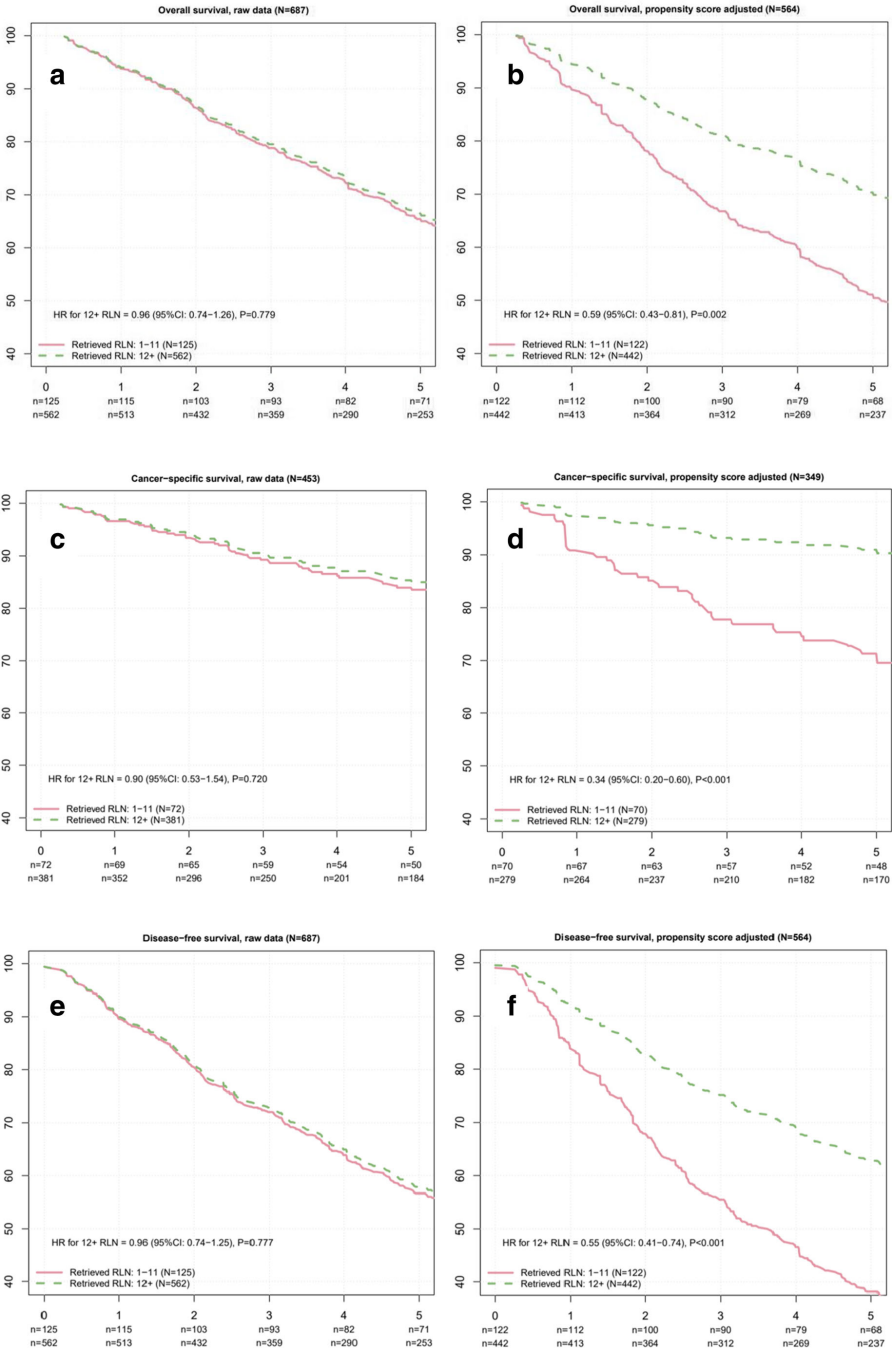
Adjusted survival curves for overall survival (**a**, **b**), cancer-specific survival (**c**, **d**) and disease-free survival (**e**, **f**). (**a**) Comparing patients with < 12 analysed LN and patients with ≥ 12 LN on OS in all stage I–III patients. (**b**) After adjustment by propensity score matching, 564 patients remain for further analysis and the effect the number of retrieved LN on OS has markedly increased. (**c**) Comparing patients with < 12 analysed LN and patients with ≥ 12 LN on CSS in all stage I–III patients. (**d**) After adjustment by propensity score matching, 349 patients remained for further analysis and the effect the number of retrieved LN on DFS has markedly increased. (**e**) Comparison of patients with < 12 patients and patients with ≥ 12 analysed LN regarding DSF in stage I–III colorectal cancer patients. (**f**) After adjustment by propensity score matching, 564 patients remain for the analysis and the effect the number of retrieved LN on DFS has markedly increased

After the matching procedure for the propensity score analysis, 5-year OS for patients with < 12 analysed LN was 53.3% (95% CI: 44.1–64.6) compared to 69.0% (95% CI: 64.4–73.8) for patients with ≥ 12 analysed LN (HR = 0.59, 95% CI: 0.43–0.81, *p* = 0.002) (Fig. 2a, b).

Five-year CSS after propensity score matching was 72.2% (95% CI: 61.9–84.3) for patients with < 12 LN compared to 89.4% (95% CI: 85.4–93.6) for patients with ≥ 12 LN (HR = 0.34, 95% CI: 0.20–0.60, *p* < 0.001) (Fig. 2c, d).

Five-year DFS after the matching procedure for the propensity score analysis for patients with < 12 LN was 41.5% (95% CI: 31.9–53.8), while it was 61.7% (95% CI: 56.8–67.0) for patients with ≥ 12 LN (HR = 0.55, 95% CI: 0.41–0.74, *p* < 0.001) (Fig. 2e, f).

### Multimodel inference for diagnostic accuracy of different cut-offs

**Fig. 3:**
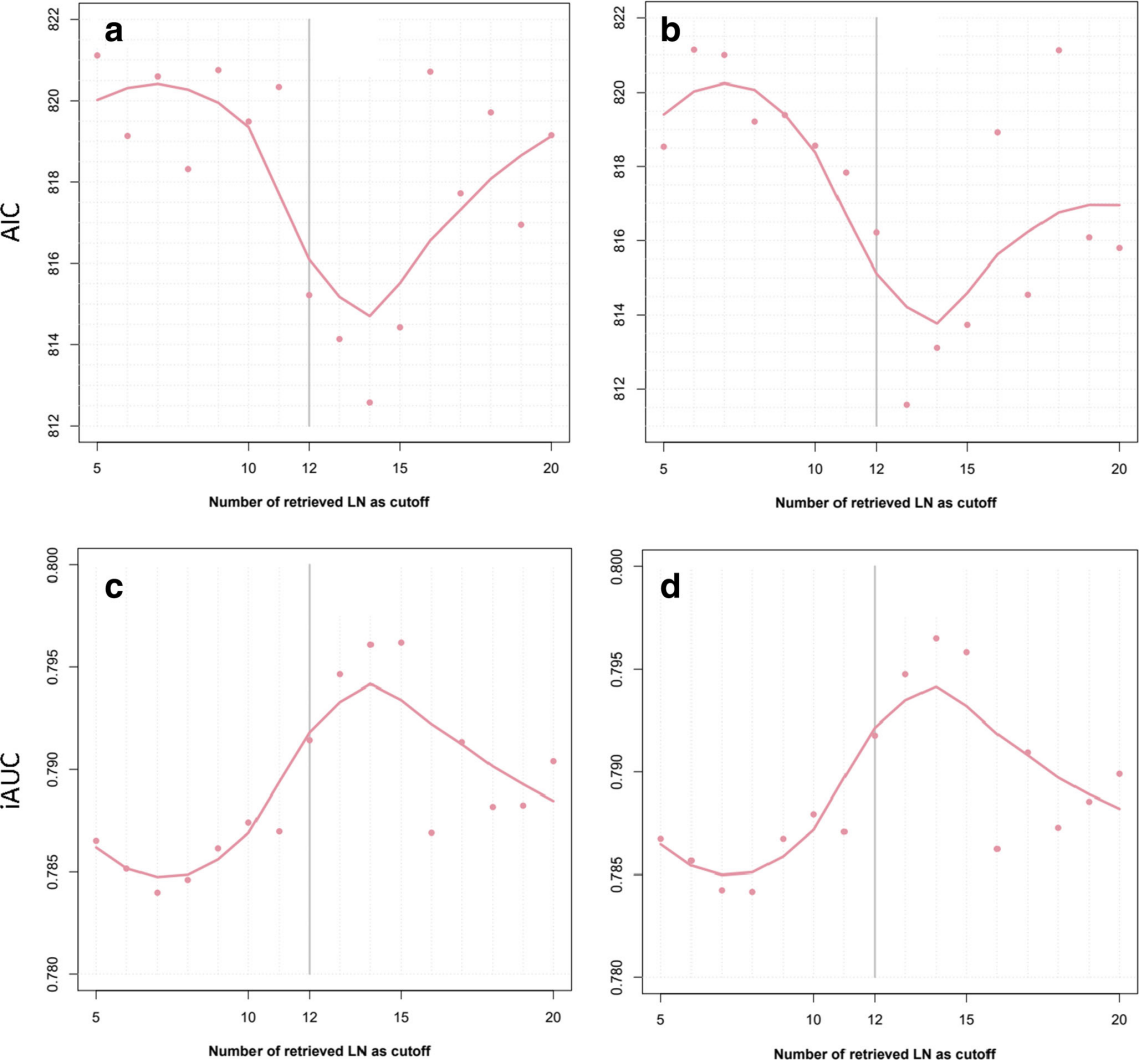
Akaike’s information criterion (AIC) for overall survival (panel **a**) and cancer specific (panel **b**) and the integrated area under the curve (iAUC) for overall survival (panel **c**) and cancer specific (panel **d**) in resampling analyses for 5 to 20 resected and analysed lymph nodes for diagnostic accuracy in multimodel inference. Lower AIC and higher iAUC indicate higher diagnostic accuracy of the cut-offs. The dots represent the observed median values, and the lines are LOESS regression lines

For OS and CSS, multimodel inference suggests more than 12 resected and analysed LN (Fig. 3). Low values for AIC and high values for iAUC indicate a better diagnostic accuracy. In terms of AIC, the statistically optimal cut-off was 14 LN for OS (AIC of 812.6) and 13 LN for CSS (median AIC of 811.6). For iAUC, the highest values were observed for 14 and 15 LN alike (median iAUC of 0.796). For DFS, inconsistent results were obtained (data not shown).

## Discussion

The recommendation to analyse at least 12 LN in patients with colorectal cancer to avoid understaging has been set relatively arbitrary, and sound evidence for this threshold is missing. By using a propensity score analysis, the here presented study demonstrates a significant improvement in OS, CSS and DFS if ≥ 12 LN are resected and analysed, thus clearly supporting the threshold of a minimum of 12 LN. The applied propensity score analysis eliminates most of the biasing factors between the two patient groups mimicking a “retrospective randomisation” of the included patients. Given the nature of the problem, to our knowledge, a standard randomised trial addressing the validity of the set threshold of 12 LN is not available, and the here used approach is probably the best way to answer this question.

Results from large series like the SEER database observed that only about 40% of patients had a sufficient number of LN examined [23, 24]. More recent results support the trend to more thorough resections and staging procedures. Numbers from a Swiss multicentre study, assessing results from operations between 2001 and 2005, were also able to obtain good quality specimens with a median number of LN of 16 (range 9–24) [25]. These results correspond with the results presented here where 82% of the 687 patients had ≥ 12 LN analysed (Table 1), and we also noted an increase in the LN yield over the years. Further, the chance for an adequate lymphadenectomy was increased in patients with a higher tumour stage and younger age. The fact that the number of resected and analysed LN significantly differed according to the year of operation is not surprising. The importance of the LN itself and the fact that not only positive LN but also the total number of LN was important had emerged only over time, and much of that awareness is attributable to several seminal publications at the time [23, 26].

However, we are not able to fully explain why the distribution of the number of resected LN is different between different stages of colorectal cancer. It might be that with early stage tumours, surgeons tend to be not as radical as with advanced stage disease, at least in the time period when the study was carried out. Another factor could be that smaller and less invasive tumours cause a less pronounced immune response what makes LN detection for pathologists more difficult. Importantly, around one-third of patients with stage I tumours had < 12 LN analysed but only about half of that proportion with stage II tumours. It is important to emphasise these results, as especially patients with a stage II tumour benefit from a proper staging and adequate adjuvant therapy if upstaged to stage III [27–30].

The number of analysed LN was considered an independent risk factor for OS, CSS and DFS in multivariate but not univariate Cox analyses (Table 2). Interestingly, only after eliminating biases between the two groups by a matching procedure for the propensity score analysis, the true impact of the number of resected and analysed LN on OS, CSS and DFS was revealed (Fig. 2). Thus, underlining the importance of a thorough analysis and an equal distribution of confounding factors before a comparison between two groups of patients is attempted.

Understaging colorectal cancer by not analysing a sufficient amount of LN has a significant impact on patients’ outcome as shown with this analysis. The risk of missing positive LN is significantly higher if < 12 are analysed. The number of resected and analysed LN has therefore become an unofficial marker for the quality of surgery, a threshold that was clearly confirmed by this study. But other factors influence the number of analysed LN as well: a dedicated team of pathologists is needed, and tumour-related factors like tumour size and stage as well as patient-related factors like age, the patients’ immune system or the amount of fat that is present in the mesentery can make retrieval of LN more difficult [28, 31–34]. For that reason, different techniques to facilitate the pathologists’ task to identify as many LN as possible were developed [35–40]. Further, sentinel LN mapping was demonstrated to improve identification of the first draining LN in the very hierarchical lymphatic draining system from the tumour. The sentinel LN has been shown to harbour tumour cells significantly more often than any other resected and analysed LN in a given specimen [41, 42].

The newest development in colon surgery on the other hand goes into a distinctly different direction: Not a more thorough analysis of a resected specimen is the highest goal but a more extensive resection including even the last LN in the draining hierarchy from a tumour [43, 44]. CME could be associated with improved survival but on the contrary also with increased morbidity [45–47].

Whether a more in-depth analysis of a specimen resected according to standard oncologic techniques, respecting plains and the anatomy or whether a more extensive resection and a standard histopathologic analysis is the future remains to be seen.

We would like to acknowledge the limitations of our study. While patients were included in the database over a period of 25 years, the follow-up period was not longer than in comparable studies. Nevertheless, we were able to detect a significant difference in regard to OS, CSS and DFS. It is estimated that more than 80% of recurrence occurs in the first 2 years after treatment, and follow-up of more than 5 years is not recommended [48]. Additionally, more than 56% of the patients were ≥ 70 years old at the time of the operation, and oncological follow-up might have been adjusted to age, and general health or postoperative surveillance, as recently demonstrated by our group, was inadequately executed [49, 50].

In summary, according to our data, the cut-off level of 12 LN seems justified. All relevant oncologic outcome measures of OS, CSS and DFS of colorectal cancer patients are significantly improved if the required number of LN are included in the specimen and analysed. The difference becomes even more obvious if all biasing factors were eliminated with a propensity score matching analysis, thereby supporting the here used statistical method if a standard randomised trial addressing the validity of the set threshold is not available. If recommendations and guidelines should be adjusted in the future, the results of our simulation suggest that a minimum number of 14 or 15 LN would yield the most significant prognostic impact (Fig. 3).

## Conclusion

Eliminating all biasing factors by a propensity score matching analysis underlines the prognostic importance of and the number of analysed lymph nodes. The set threshold marks the minimum number of required LN but nevertheless represents a cut-off regarding outcome in stage I–III CRC. This analysis therefore highlights the significance and importance of adherence to surgical oncological standards and suggests an adaptation of the number of minimally required lymph nodes for future guidelines.
